# Supplementary material for: Immunogenomic engineering of a plug-and-(dis)play hybridoma platform
Source: Nat Commun. 2016 Aug 17;7:12535. doi: 10.1038/ncomms12535 (PMC4992066; doi:10.1038/ncomms12535)
Supplement: Supplementary Information — Supplementary Figures 1-9 and Supplementary Tables 1-4 [file ncomms12535-s1.pdf]

a

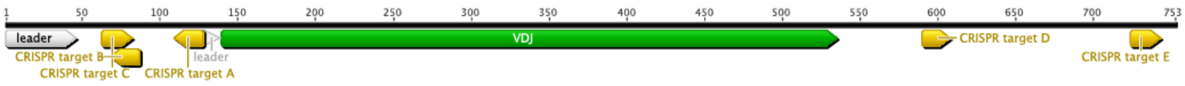

Leader peptide
Antibody variable region
CRISPR gRNA target site
PAM

Nucleotide sequence

ATGGAATGGCCTTGTATCTTTCTCCTCCTCCTGTCAGTCACTGAAGGTAAGGAACTCAGCAGTTCCAATCTGAAGAGGAGATAGGGCCT
GAGGTGACAATGACATCCACAATTTCTTTCTCCCGACAGGTGTCCACTCCCAGGTTCAGCTGCAGCAGTCTGGACCTGAGCTGGTGAA
GCCTGGGGCCTCAGTGAAGATTTCCTGCAAGGCTTCTGGCTACGCATTCAGTAACTCCTGGATGAACTGGGTGAAGCAGAGGCCTG
GAAAGGGTCTTGAGTGGATTGGACGGATTTATCCTGGAGATGGAGATACTAACTACAATGGGAAGTTCAGGGGCAAGGCCACACTG
ACTGCAGACAAATCCTTCAGCACAGCCTACATACAACTCAGCAGCCTGACATCTGAGGACTCTGCGGTCTACTTCTGCGCAAGAAAT
ATCGTTGGTGAAGGTCTCTACTATGATTACGACGGGGGGGAACTTTTTGACTATACTGTGGACTGCTGGGGTCAAGGAACCTCAGTC
ACCGTCTCCTCAGGTATGAATGGCCTCTCCAGGTCTTTACTTTTCACCTTTCTTATGGGGTTTTCTGAGCATGACGGACTAATCTTGGATATTT
GTCCCTGAGGGAGCCGGCTGAGAAAAATGGTAAATAAACTGTCTAGGGATCTCTGAGCCTTTAGGACAGATTATCTCCACATCTTTGAAAAA
CTAAGAGTCTGTGTGTTGGTTTTAGCGGAGTCCCTGGATGATGG

Translated ORF

MEWPCIFLLLLSVTEGVHSQVQLQQSGPELVKPGASVKISCKASGYAFSNSWMNWVKQRPGKGLEWIGRIYPGDGDTNYNGKFRGKATL
TADKSFSTAYIQLSSLTSEDSAVYFCARNIVGEGLYYDYDGGELFDYTVDCWGQGTSVTVSS

b

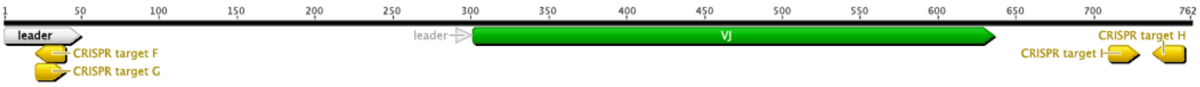

Leader peptide
Antibody variable region
CRISPR gRNA target site
PAM

ATGGAGAAAGACACACTCCTGCTATGGGTCCTGCTTCTCTGGGTTCCAGGTGAGGATGCAGAGAAGTGTTGAGAGCAACCTCTGTAG
CCATCATGACTTTCCATGTTTCTGGATTCTTGATCATTATACTTAGGGTATTTGTCATTGGTTTTAAGATTCCTCAGTCCCCTGGATTTTCT
GGTTTCTCAGAGTGATATCCACAGTCATTCTTAAAATTTTAAACTGAGAGGTCCTCTGCTGGGAAGGTATGTCCACATACATGACAATAG
CCTGTGTGTTTCTCATTCCAGGTTCCACAGGTGACATTGTGCTGACCCAATCTCCAGCTTCTTTGGCTGTGTCTCTAGGGCAGAGGG
CCACCATCTCCTGCAGAGCCAGCGAGAGTGTTTATAATTATGGCTTTAGTTTTATGAGCTGGTTCCAACAAAAACCAGGACAGCCAC
CCAAGCTCCTCATCTATGTTGCATCCAACCAAGGATCCGGGGTCCCTGCCAGGTTTAGTGGCAGTGGGTCTGGGACAGACTTCAGC
CTCAACATCCATCCTGTGGAGGAGGATGATACTGCAATGTATTTCTGTCAACAAACTAAGGAGGTTCCATTCACGTTCGGCTCGGGG
ACAAAGTTGGAAATAAAACGTAAGTAGACTTTTGCTCATTTACTTGTGACGTTTTGGTTCTGTTTGGGTAACTTGTGTGAATTTGTGACA
TTTTGGCTAAATGAGCCATTCCTGGCAACCTGTGCATCAATAGAAGATCCCCCAGA

Translated ORF

MEKDTLLLWVLLLWVPGSTGDIVLTQSPASLAVSLGQRATISCRASESVYNYGFSFMSWFQQKPGQPPKLLIYVASNQGSGVPARFSGSG
SGTDFSLNIHPVEEDDTAMYFCQQTKEVPFTFGSGTKLEIK

Supplementary Fig. 1. Annotated genomic sequence of immunoglobulin loci in WT hybridoma cells. (**a**) Genomic sequence of the IgH locus. (**b**) Genomic sequence of the IgK locus. Translated ORF were predicted from the gDNA and cDNA sequences. CRISPR gRNA target sites were independently tested (see **Supplementary Fig. 2**).

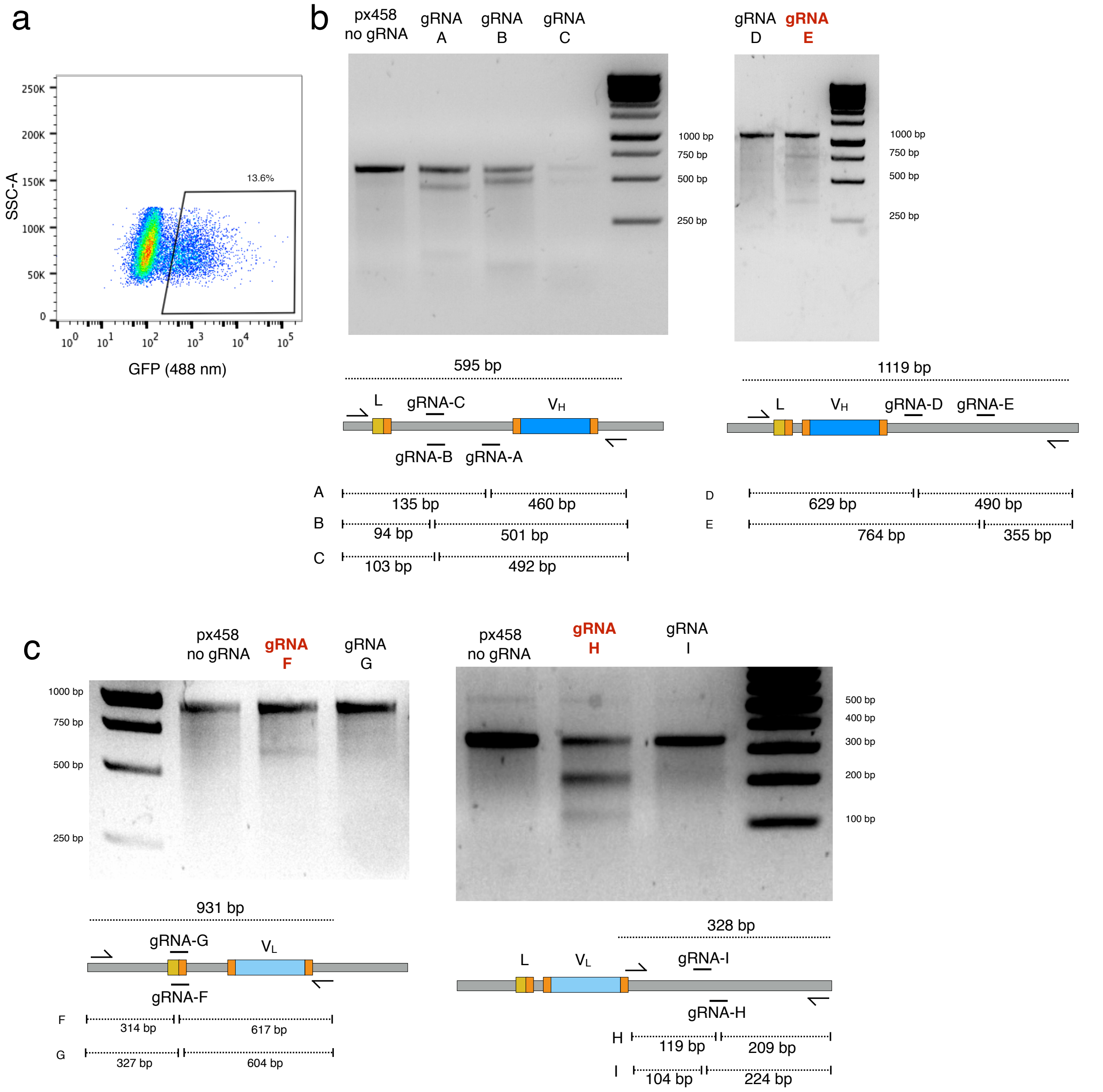

Supplementary Fig. 2. Validation of CRISPR-Cas9 targeting of immunoglobulin loci in WT hybridoma cells. (**a**) Flow cytometry dot plot shows expression of Cas9 (via 2A-GFP) in WT cells following transfection with pX458. (**b, c**) Surveyor results validate CRISPR-Cas9 targeting in IgH and IgK loci, respectively (agarose gels of all gRNA sites tested). The gRNAs selected for further experiments are in red text.

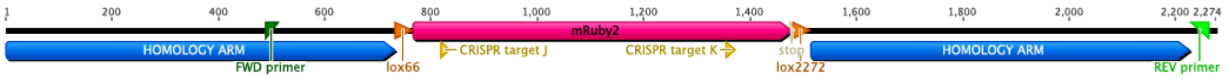

mRuby2 gene
Homology Arm
CRISPR gRNA target site
PAM
LoxP sites
Forward sequencing primer
Reverse sequencing primer
lowercase is external to genomic region

Nucleotide sequence

AGTTATATCTAATTCCATTCATTTTTTCCAGAATCAGTGTAAATTTCTGGTAAAAATCATTTATTTGAAATCTATGACTTAGTGATAAAATCCTTTGTTG
ACACATCTATAGTTTATATCTATAATTTAGTTAATCATGGTATTGGTGTAATAATCACTGATGTGTAAATATACATGTACTTTTATCCATTTTAGTGAATT
TTGAAGTACATAACCTGTGGTAAAGGCAAATATTACTTGAACGTATCTTAAAGAAGGTTCTAATATCTAAAGTTAGCCACTTTTGGAATTTACCAT
CAGATCATCATCATCATCATCATCATCATCATCATCATCATCATCATGATGCTCACAAAGCACCATCGCTAAATCCATGTGTTAATATTGTCCTAAA
GTGGAGCAGCAGACTCAGGCCAAAAATTTATTGAGAACTTAGTCCCTGAAGTTACATCCACAACATCTGGCCAGGGCTCAGGGAAAGTGTT
TGG**GATGCTTTTCCTCAGGGAGGATTATG**ACTTGGACCCTAGCATCCTGCTGCATGACCCATGTGCCTTTTCAGTGCTTTCTCCCTAGTTCT
TCTCCAGCTGGACTAGGTCATTAACTAAGAAATGCACTGCTCATGAATATGCAAATTACTTGAGCCTATGGTAGTAAATACAGGCATGCCCACA
CTGTGAAAACAACATATGACTCCTGTCTTCTCCACAGTCCCAGAACACACTCACTCTAACC**ATAACTTCGTATAATGTATGCTATACGAACG**
GTAATGGTGTCTAAGGGCGAAGAGCTGATCAAGGAAAATATGCGTATGAAGGTG*GTCATGGAAGGTTCGGTCAACGG*CCACCAATTC
AAATGCACAGGTGAAGGAGAAGGCAATCCGTACATGGGAACTCAAACCATGAGGATCAAAGTCATCGAGGGAGGACCCCTGCCATT
TGCCTTTGACATTCTTGCCACGTCGTTCATGTATGGCAGCCGTACTTTTATCAAGTACCCGAAAGGCATTCCTGATTTCTTTAAACAGTC
CTTTCCTGAGGGTTTTACTTGGGAAAGAGTTACGAGATACGAAGATGGTGGAGTCGTCACCGTCATGCAGGACACCAGCCTTGAGGA
TGGCTGTCTCGTTTACCACGTCCAAGTCAGAGGGGTAAACTTTCCCTCCAATGGTCCCGTGATGCAGAAGAAGACCAAGGGTTGGGA
GCCTAATACAGAGATGATGTATCCAGCAGATGGTGGTCTGAGGGGATACACTCATATGGCACTGAAAGTTGATGGTGGTGGCCATCTG
TCTTGCTCTTTCGTAACAACTTACAGGTCAAAAAAGACCGTCGGGAACATCAAGATGCCCGGTATC*CATGCCGTTGATCACCGCCTGG*
AAAGGTTAGAGGAAAGTGACAATGAAATGTTCGTAGTACAACGCGAACACGCAGTTGCCAAGTTCGCCGGGCTTGGTGGTGGGATG
GACGAGCTGTACAAGTGAATAACTTC**GTATAAAGTATCCTATACGAAGTTAT**TCACCGTCTCCTCAGGTAAGAATGGCCTCTCCAGGTCTT
TATTTTTAACCTTTGTTATGGAGTTTTCTGAGCATTGCAGACTAATCTTGGATATTTGTCCCTGAGGGAGCCGGCTGAGAGAAGTTGGGAAATA
AACTGTCTAGGGATCTCAGAGCCTTTAGGACAGATTATCTCCACATCTTTGAAAAACTAAGAATCTGTGTGATGGTGTTGGTGGAGTCCCTGG
ATGATGGGATAGGGACTTTGGAGGCTCATTTGAAGAAGATGCTAAAACAATCCTATGGCTGGAGGGATAGTTGGGGCTGTAGTTGGAGATTTT
CAGTTTTTAGAATAAAAGTATTAGTTGTGGAATATACTTCAGGACCACCTCTGTGACAGCATTTATACAGTATCCGATGCATAGGGACAAAGAGT
GGAGTGGGGCACTTTCTTTAGATTTGTGAGGAATGTTCCGCACTAGATTGTTTAAAACTTCATTTGTTGGAAGGAGAGCTGTCTTAGTGATTGA
GTCAAGGGAGAAAGGCATCTAGCCTCGGTCTCAAAAGGGTAGTTGCTGTCTAGAGAGGTCTGGTGGAGCCTGCAAAAGTCCAGCTTTCAA
AGGAACACAGAAGTATGTGTATGGAATATTAGAAGATGTTGCTTTTACTCTTAAGTTGGTTCCTAGGAAAAATAGTTAAATACTGTGACTTTAAAA
TGTGAGAGGGTTTTCAAgta**ctcattttttaaatgtccaaaattcttgtcaatca**

mRuby2 coding sequence

MVSKGEELIKENMRMKVVMEGSVNGHQFKCTGEGEGNPYMGTQTMRIKVIEGGPLPFAFDILATSFMYGSRTFIKYPKGIPDFFKQSFPEG
FTWERVTRYEDGGVVTVMQDTSLEDGCLVYHVQVRGVNFPSNGPVMQKKTKGWEPNTEMMYPADGGLRGYTHMALKVDGGGHLSCSF
VTTYRSKKTVGNIKMPGIHAVDHRLERLEESDNEMFVVQREHAVAKFAGLGGGMDELYK

Supplementary Fig. 3. Annotated sequence of PnP-mRuby locus. PCR performed on genomic DNA from PnP-mRuby cells (clone 1E9.C3, see **Supplementary Table 1**) followed by Sanger sequencing (directly on PCR product or following bacterial cloning) verified matching to reference sequence from nucleotide position 489 to 2264. CRISPR gRNA target sites were independently tested (see **Supplementary Fig. 6**).

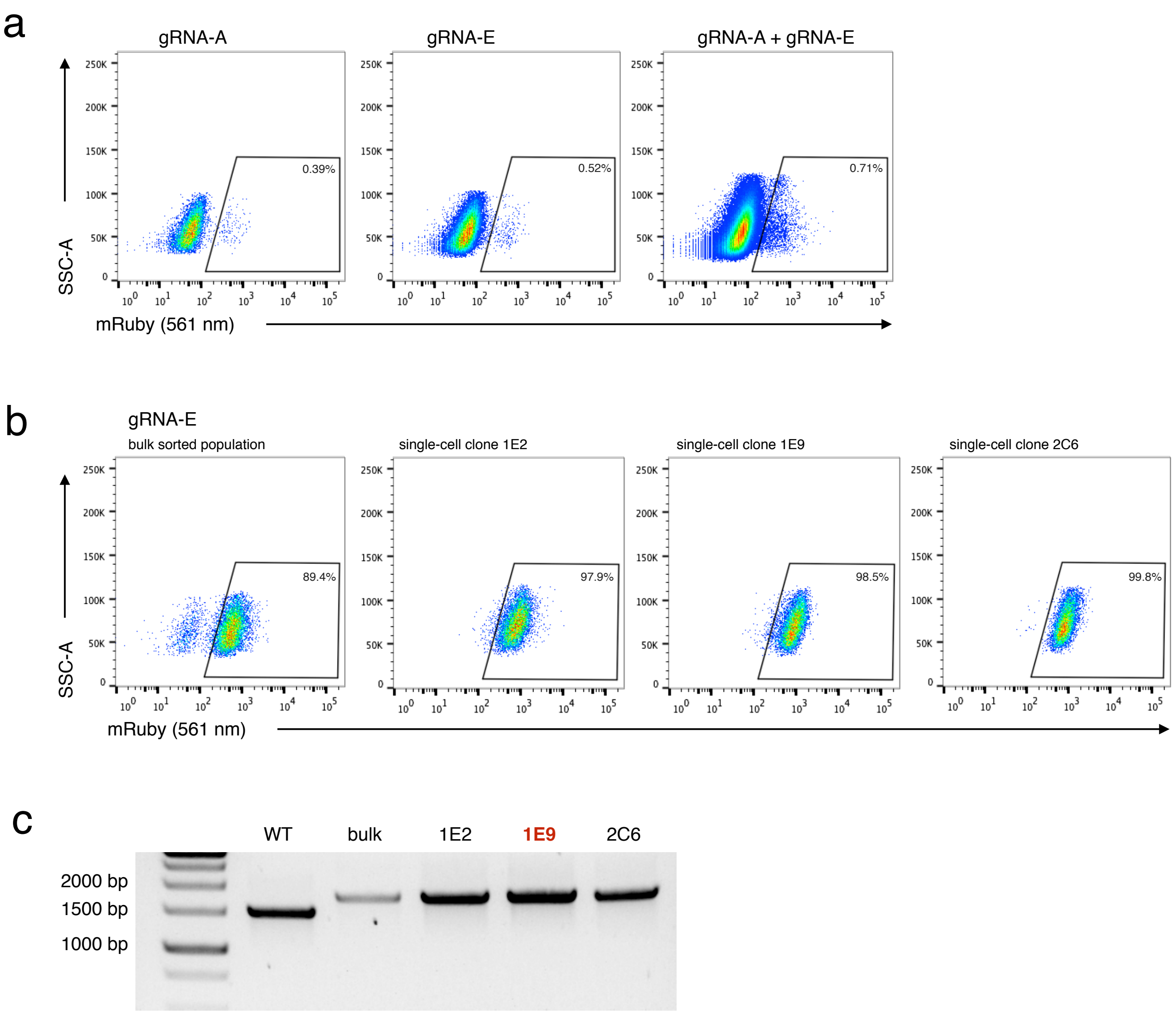

Supplementary Fig. 4. Generation and characterization of PnP-mRuby clones. (**a**) Flow cytometry dot plots following transfection WT cells with pX458 (with different gRNAs) and mRuby donor construct (linear). Data is before sorting for mRuby expression. (**b**) Flow cytometry dot plots after sorting for mRuby expression in different samples, before (bulk sorted population) or after the final single-cell sorting step. (**c**) DNA agarose gels of PCR on genomic DNA using the same primers shown in **Fig. 1d**. Clone 1E9 was selected for further experiments.

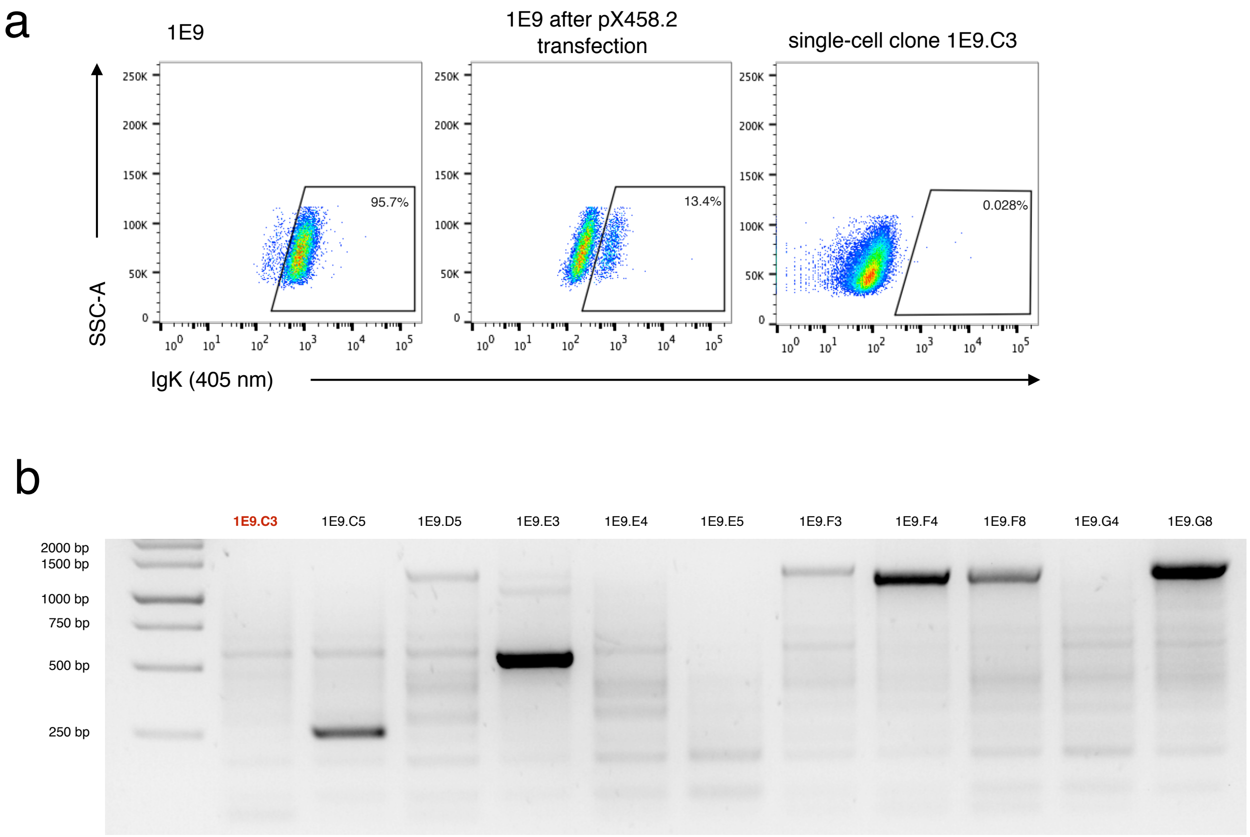

Supplementary Fig. 5. Characterization of PnP-mRuby cells with deletion of V_L. (**a**) Left panel, flow cytometry dot plot shows IgK expression is still present in clone 1E9 (mRuby integrated in IgH locus). Middle panel shows 1E9 cells following transfection with pX458 with gRNAs targeting V_L. Right panel shows single-cell isolated clone 1E9.C3 with nearly all IgK expression knocked out. (**b**) DNA agarose gel of PCRs from single-cell sorted populations from middle panel of (A). Primers used for PCR are the same shown in **Fig. 1g**.

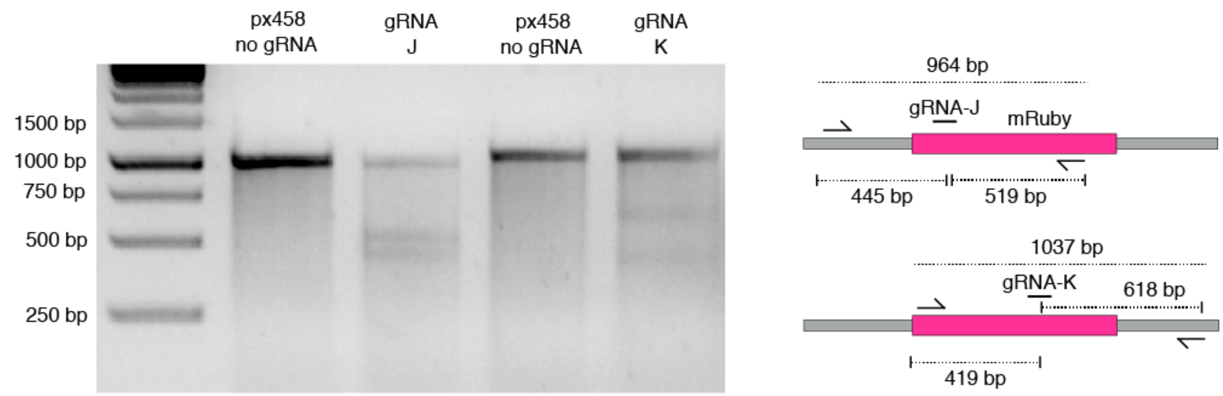

Supplementary Fig. 6. Validation of CRISPR-Cas9 targeting in mRuby gene. Surveyor assays following transfection of PnP-mRuby cells (clone 1E9.C3) with pX458.2 and gRNAs targeting the mRuby gene.

a

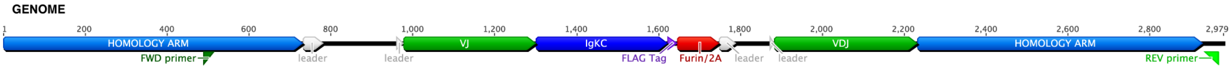

Leader peptide
Antibody variable region
Antibody constant region
FLAG tag

Furin/2A peptide
Homology arms
Forward sequencing primer
Reverse sequencing primer (lowercase)

Nucleotide sequence

AGTTATATCTAATTCCATTCATTTTTTCCAGAATCAGTGTAAATTTCTGGTAAAAATCATTTATTTGAAATCTATGACTTAGTGATAAAATCCTTTGTTGACACATCTATAGTTTATATCTATAATTTAGTTAATCATGGTATTGGTGTAATAATCACTGATGTG
TAAATATACATGTACTTTTATCCATTTAGTGAATTTTGAAGTACATAACCTGTGGTAAAGGCAAATATTACTTGAACGTATCTTAAAGAAGGTTCTAATATCTAAAGTTAGCCACTTTTGGAATTTACCATCAGATCATCATCATCATCATCATCATC
ATCATCATCATCATCATGATGCTCACAAAGCACCATCGCTAAATCCATGTGTTAATATTGTCCTAAAGTGGAGCAGCAGACTCAGGCCAAAAATTTATTGAGAACTTAGTCCCTGAAGTTACATCCACAACATCTGGCCAGGGCTCAGGGAAAG
TGTTTGG*GATGCTTTTCCTCAGGGAGGATTATGA*CTTGGACCCTAGCATCCTGCTGCATGACCCATGTGCCTTTTCAGTGCTTTCTCCCTAGTTCTTCTCCAGCTGGACTAGGTCATTAACTAAGAAATGCACTGCTCATGAATATGCAAATTA
CTTGAGCCTATGGTAGTAAATACAGGCATGCCCACACTGTGAAAACAACATATGACTCCTGTCTTCTCCACAGTCCCAGAACACACTCACTCTAACCATGGATTTTCAGGTGCAGATTTTCAGCTTCCTGCTAATCAGTGCCTCAGGTAAC
AGAGGGCAGGGAATTTGAGATCAGAATCCAGCCAGAATTATTTTCCCTGGGGAATTTGAGTCTAAAATACAGTTTTTTTTTTCTTTTTCTTTCATCTGAATGTTAGGTAGTATAAAATTATTTTTGCATCTCTATTTCTACTAATCCCTCTCTCTCTATTT
TCTTTTTTCTAGTCATAATGTCCAGAGGAGACATTGTGCTGACACAGTCTCCAGCAATCATGTCTGCATCTCTAGGGGAACGGGTCACCATGACCTGCACTGCCAGCTCAAATGTAAGTTCCAGTTACTTGCACTGGTACCAGCAG
AAGCCAGGATCCTCCCCCAAACTCTGGATTTATAGCACATCCAACCTGGCTTCTGGAGTCCCAGCTCGCTTCAGTGGCAGTGGGTCTGGGACCTCTTACTCTCTCACAATCAGCAGCATGGAGGCTGAAGATGCTGCCACTTATT
ACTGCCACCAGTATCATCGTTCCCCGCCCACGTTCGGTGCTGGGACCAAGCTGGAAATCAAACGGGCTGATGCTGCACCAACTGTATCCATCTTCCCACCATCCAGTGAGCAGTTAACATCTGGAGGTGCCTCAGTCGTGTGCT
TCTTGAACAACTTCTACCCCAAAGACATCAATGTCAAGTGGAAGATTGATGGCAGTGAACGACAAAATGGCGTCCTGAACAGTTGGACTGATCAGGACAGCAAAGACAGCACCTACAGCATGAGCAGCACCCTCACGTTGACC
AAGGACGAGTATGAACGACATAACAGCTATACCTGTGAGGCCACTCACAAGACATCAACTTCACCCATTGTCAAGAGCTTCAACAGGAATGAGTGTGACTACAAGGATGACGACGATAAG*AGGAAAAGACGACACAAACAGAA*
*AATTGTGGCACCGGTGAAACAGACTTTGAATTTTGACCTTCTCAAGTTGGCGGGAGACGTCGAGTCCAACCCTGGGCCC*ATGATGGTGTTAAGTCTTCTGTACCTGTTGACAGCCCTTCCGGGTGAGTGTTTCCATTTCATACATGT
GCCATGAGGATTTTTCAAAATGTGTGATTGACAGATTTGATTCTTTTTGTCTAAAGGTATCCTGTCAGAAGTGCAGCTGCAGCAGAGCGGCGCGGAACTGATGAAACCGGGCGCGAGCGTGAAAATTAGCTGCAAAGCGACCGGCTAT
ACCTTTAGCAACTATTGGATTGGCTGGGTGAAACAGCGTCCGGGCCATGGCCTGGAATGGATTGGCGAAATTCTGCCGGGCAGCGGCAGCACCAACTATAACGAAAAATTTAAAGGCAAAGCGACCTTTACCGCGGATACCAG
CAGCAACACCGCGTATATGCAGCTGAGCAGCCTGACCAGCGAAGATAGCGCGGTGTATTATTGCGCGCGTGATAGCAGCGGCGGCTTTGCGTATTGGGGCCAGGGCACCCTGGTGACCGTGAGCGCGGGTAAGAATGGCCTCT
CCAGGTCTTTATTTTTAACCTTTGTTATGGAGTTTTCTGAGCATTGCAGACTAATCTTGGATATTTGTCCCTGAGGGAGCCGGCTGAGAGAAGTTGGGAAATAAACTGTCTAGGGATCTCAGAGCCTTTAGGACAGATTATCTCCACATCTTTGA
AAAACTAAGAATCTGTGTGATGGTGTTGGTGGAGTCCCTGGATGATGGGATAGGGACTTTGGAGGCTCATTTGAAGAAGATGCTAAAACAATCCTATGGCTGGAGGGATAGTTGGGGCTGTAGTTGGAGATTTTCAGTTTTTAGAATAAAAGTA
TTAGTTGTGGAATATACTTCAGGACCACCTCTGTGACAGCATTTATACAGTATCCGATGCATAGGGACAAAGAGTGGAGTGGGGCACTTTCTTTAGATTTGTGAGGAATGTTCCGCACTAGATTGTTTAAAACTTCATTTGTTGGAAGGAGAGCT
GTCTTAGTGATTGAGTCAAGGGAGAAAGGCATCTAGCCTCGGTCTCAAAAGGGTAGTTGCTGTCTAGAGAGGTCTGGTGGAGCCTGCAAAAGTCCAGCTTTCAAAGGAACACAGAAGTATGTGTATGGAATATTAGAAGATGTTGCTTTTAC
TCTTAAGTTGGTTCCTAGGAAAAATAGTTAAATACTGTGACTTTAAAATGTGAGAGGGTTTTCAA*gtactcatttttttaaatgtccaaaattcttgtcaatca*

b

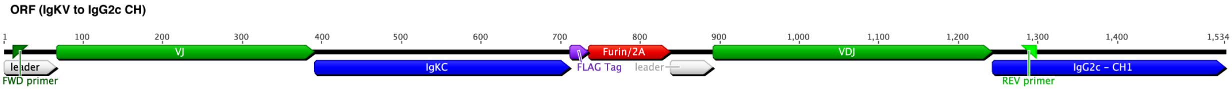

Leader peptide
Antibody variable region
Antibody constant region
FLAG tag

Furin/2A peptide
Forward sequencing primer
Reverse sequencing primer
lowercase is external to genomic region

Nucleotide sequence

ATGGATTTTCA*GGTGCAGATTTTCAGCTTC*CTGCTAATCAGTGCCTCAGTCATAATGTCCAGAGGAGACATTGTGCTGACACAGTCTCCAGCAATCATGTCTGCATCTCTAGGGGAACGGGTCACCATGACCTGCACTGCCAGCTC
AAATGTAAGTTCCAGTTACTTGCACTGGTACCAGCAGAAGCCAGGATCCTCCCCCAAACTCTGGATTTATAGCACATCCAACCTGGCTTCTGGAGTCCCAGCTCGCTTCAGTGGCAGTGGGTCTGGGACCTCTTACTCTCTCACA
ATCAGCAGCATGGAGGCTGAAGATGCTGCCACTTATTACTGCCACCAGTATCATCGTTCCCCGCCCACGTTCGGTGCTGGGACCAAGCTGGAAATCAAACGGGCTGATGCTGCACCAACTGTATCCATCTTCCCACCATCCAGTG
AGCAGTTAACATCTGGAGGTGCCTCAGTCGTGTGCTTCTTGAACAACTTCTACCCCAAAGACATCAATGTCAAGTGGAAGATTGATGGCAGTGAACGACAAAATGGCGTCCTGAACAGTTGGACTGATCAGGACAGCAAAGACA
GCACCTACAGCATGAGCAGCACCCTCACGTTGACCAAGGACGAGTATGAACGACATAACAGCTATACCTGTGAGGCCACTCACAAGACATCAACTTCACCCATTGTCAAGAGCTTCAACAGGAATGAGTGTGACTACAAGGATG
ACGACGATAAG*AGGAAAAGACGACACAAACAGAAAATTGTGGCACCGGTGAAACAGACTTTGAATTTTGACCTTCTCAAGTTGGCGGGAGACGTCGAGTCCAACCCTGGGCCC*ATGATGGTGTTAAGTCTTCTGTACCTGTTGA
CAGCCCTTCCGGGTATCCTGTCAGAAGTGCAGCTGCAGCAGAGCGGCGCGGAACTGATGAAACCGGGCGCGAGCGTGAAAATTAGCTGCAAAGCGACCGGCTATACCTTTAGCAACTATTGGATTGGCTGGGTGAAACAGCGT
CCGGGCCATGGCCTGGAATGGATTGGCGAAATTCTGCCGGGCAGCGGCAGCACCAACTATAACGAAAAATTTAAAGGCAAAGCGACCTTTACCGCGGATACCAGCAGCAACACCGCGTATATGCAGCTGAGCAGCCTGACCAG
CGAAGATAGCGCGGTGTATTATTGCGCGCGTGATAGCAGCGGCGGCTTTGCGTATTGGGGCCAGGGCACCCTGGTGACCGTGAGCGCGGCCAAAACAACAGCCCCATCGGTCTATCCACTGGCCC*CTGTGTGTGGAGGTACAA*
*C*TGGCTCCTCGGTGACTCTAGGATGCCTGGTCAAGGGTTATTTCCCTGAGCCAGTGACCTTGACCTGGAACTCTGGATCCCTGTCCAGTGGTGTGCACACCTTCCCAGCTCTCCTGCAGTCTGGCCTCTACACCCTCAGCAGCT
CAGTGACTGTAACCTCGAACACCTGGCCCAGCCAGACCATCACCTGCAATGTGGCCCACCCGGCAAGCAGCACCAAAGTGGACAAGAAAATTGAGCCCAGAGTGCCCATAACACAGAACCCCTGTCCTCCACTCAAAGAGTG
TCCCCCATGCGCAGCTCCAGACCTCTTGGGTGGACCATCCGTCTTCATCTTCCCTCCAAAGATCAAGGATGTACTCATGATCTCCCTGAGCCCCATGGTCACATGTGTGGTGGTGGATGTGAGCGAGGATGACCCAGACGTCCA
GATCAGCTGGTTTGTGAACAACGTGGAAGTACACACAGCTCAGACACAAACCCATAGAGAGGATTACAACAGTACTCTCCGGGTGGTCAGTGCCCTCCCCATCCAGCACCAGGACTGGATGAGTGGCAAGGAGTTCAAATGCA
AGGTCAACAACAGAGCCCTCCCATCCCCCATCGAGAAAACCATCTCAAAACCCAGAGGGCCAGTAAGAGCTCCACAGGTATATGTCTTGCCTCCACCAGCAGAAGAGATGACTAAGAAAGAGTTCAGTCTGACCTGCATGATC
ACAGGCTTCTTACCTGCCGAAATTGCTGTGGACTGGACCAGCAATGGGCGTACAGAGCAAAACTACAAGAACACCGCAACAGTCCTGGACTCTGATGGTTCTTACTTCATGTACAGCAAGCTCAGAGTACAAAAGAGCACTTGG
GAAAGAGGAAGTCTTTTCGCCTGCTCAGTGGTCCACGAGGGTCTGCACAATCACCTTACGACTAAGACCATCTCCCGGTCTCTGGGAAAA

c

Leader peptide
Antibody variable region
Antibody constant region
FLAG tag

ORF (IgKV to IgG2c CH) translation

MDFQVQIFSFLLISASVIMSRGDIVLTQSPAIMSASLGERVTMTCTASSNVSSSYLHWYQQKPGSSPKLWIYSTSNLASGVPARFSGSGSGTSYSLTISSMEAEDAATYYCHQYHRSPPTFGAGTKLEIKRADAAPTVSIFPPSSEQLTSGGA
S V V C F L N N F Y P K D I N V K W K I D G S E R Q N G V L N S W T D Q D S K D S T Y S M S S T L T L T K D E Y E R H N S Y T C E A T H K T S T S P I V K S F N R N E C D Y K D D D D K - -
MMVLSLLYLLTALPGILSEVQLQQSGAELMKPGASVKISCKATGYTFSNYWIGWVKQRPGHGLEWIGEILPGSGSTNYNEKFKGKATFTADTSSNTAYMQLSSLTSEDSAVYYCARDSSGGFAYWGQGTLVTVSAAKTTAPSVYPLAPVC
GGTTGSSVTLGCLVKGYFPEPVTLTWNSGSLSSGVHTFPALLQSGLYTLSSSVTVTSNTWPSQTITCNVAHPASSTKVDKKIEPRVPITQNPCPPLKECPPCAAPDLLGGPSVFIFPPKIKDVLMISLSPMVTCVVVDVSEDDPDVQISWFVN
NVEVHTAQTQTHREDYNSTLRVVSALPIQHQDWMSGKEFKCKVNNRALPSPIEKTISKPRGPVRAPQVYVLPPPAEEMTKKEFSLTCMITGFLPAEIAVDWTSNGRTEQNYKNTATVLDSDGSYFMYSKLRVQKSTWERGSLFACSVVHE
GLHNHLTTKTISRSLGK

Supplementary Fig. 7. Genomic characterization of PnP-HEL23 cells. (**a**) Genomic sequence of the IgH locus in PnP-HEL23 cells with integrated sAb constructs. PCR and cloning was performed on genomic DNA, which was followed by Sanger sequencing, which verified consensus agreement to reference sequence from nucleotide positions 489 to 2912. (**b**) Sequence of the unique transcript coding the full-length antibody, from the leader of V_L to the first exon of the IgH, CH_1. RT-PCR performed on mRNA followed by Sanger sequencing verified correct splicing and matching to reference sequence from nucleotide positions 21 to 1288. (**c**) Predicted amino acid sequence of the translated ORF. The two dashes (-) in red indicate the ribosomal skipping and furin cleavage site (Furin/2A). The PnP-HEL23 sequence throughout the figure corresponds to the consensus between clones Y and AC (see **Supplementary Table 1**).

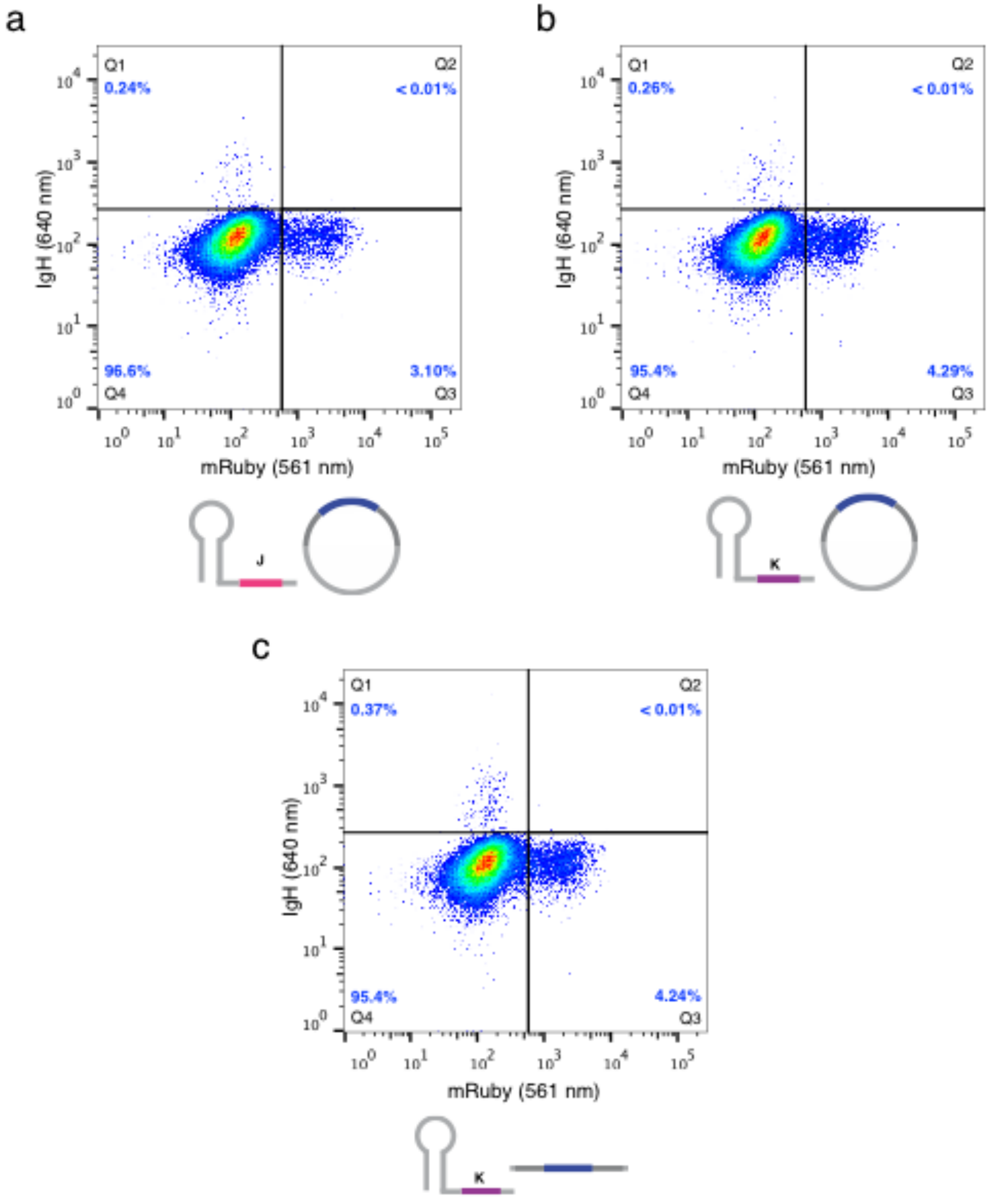

Supplementary Fig. 8. Evaluation of gRNA and HDR donor formats for generation of PnP-HEL23 cells. Different gRNAs and HDR donor formats were evaluated for the generation PnP-HEL23 cell lines. Flow cytometry dot plots correspond to the pre-IgH sorting phase, analogous to **Fig. 2d,** which displays the format with highest efficiency (gRNA-J, linear HDR donor). (**a**) gRNA-J, plasmid HDR donor. (**b**) gRNA-K, plasmid HDR donor. (**c**) gRNA-K, linear HDR donor.

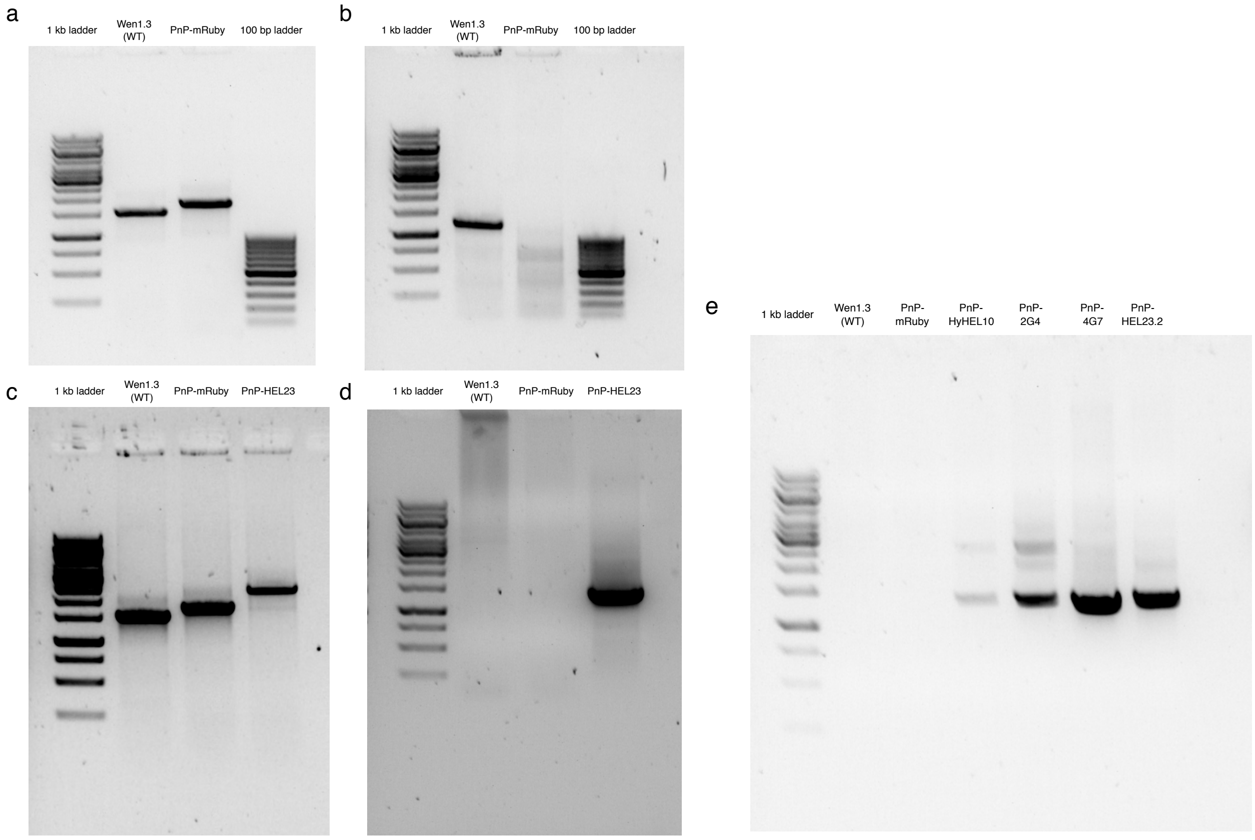

Supplementary Fig. 9. Uncropped DNA agarose gel pictures. (**a**) Genomic V_H locus from WT hybridoma and engineered PnP-mRuby cell line (ref.: **Fig. 1d**). (**b**) Genomic V_L locus from WT hybridoma and engineered PnP-mRuby cell line (ref.: **Fig. 1g**). (**c**) Genomic V_H locus from WT hybridoma, PnP-mRuby and PnP-HEL23 reprogrammed cell lines (ref.: **Fig. 2g**). (**d**) Spliced HEL23 synthetic antibody (sAb) amplified by RT-PCR (ref.: **Fig. 2h**). (**e**) Spliced HyHEL10, 2G4, 4G7 and HEL23.2 sAbs amplified by RT-PCR (ref.: **Fig. 4f**).

Table S1. Summary of hybridoma clones used and generated in this study.

Cell Name	Cell line clone	Description
WT	WEN1.3	WEN1.3 cells are derived from a mouse infected with LCMV. They express IgG2c and are specific for LCMV GP-1 antigen. Their IgH and IgK loci were sequenced and annotated (as shown in Fig. S1)
PnP-mRuby	1E9.C3	WEN1.3 cells were transfected with pX458 with gRNA-E and mRuby donor construct and sorted for Cas9 positive expression (2A-GFP). This was followed by a first round of sorting for mRuby-positive cells, followed by a second single-cell sort for mRuby. A single-cell clone was selected and then transfected with pX458 with gRNA-F and H and sorted for Cas9 positive expression (2A-GFP). Cells were single-cell sorted for IgK negative expression, followed by genomic PCR to identify a clone with V_L deletion. This final clone represents 1E9.C3
PnP-HEL23	Y	Clone 1E9.C3 was transfected with pX458.2 with gRNA-J and HEL23 sAb donor (linearized). Cells were sorted for Cas9 positive expression (2A-BFP) and expanded. Cells were then sorted for surface IgH expression and expanded, and finally characterized for IgH and IgK expression.
PnP-HyHEL10	U	Clone 1E9.C3 was transfected with pX458.2 with gRNA-J and HyHEL10 sAb donor (linearized). Cells were sorted for Cas9 positive expression (2A-BFP) and expanded. Cells were then sorted for surface IgH expression and expanded, and then they underwent a second IgH sort. They were finally characterized for IgH and IgK expression.
PnP-2G4	AA	Clone 1E9.C3 was transfected with pX458.2 with gRNA-J and 2G4 sAb donor (linearized). Cells were sorted for Cas9 positive expression (2A-BFP) and expanded. Cells were then sorted for surface IgH expression and expanded, and then they underwent a third sort for IgH and IgK expression. They were finally characterized for IgH and IgK expression.
PnP-4G7	AB	Clone 1E9.C3 was transfected with pX458.2 with gRNA-J and 4G7 sAb donor (linearized). Cells were sorted for Cas9 positive expression (2A-BFP) and expanded. Cells were then sorted for surface IgH expression and expanded, and then they underwent a third sort for IgH and IgK expression. They were finally characterized for IgH and IgK expression.
PnP-HEL23.2	AC	Clone 1E9.C3 was transfected with pX458.2 with gRNA-J and HEL23 sAb donor (linearized). Cells were **not** sorted for Cas9 positive expression. They were sorted only once for surface IgH expression and expanded. They were finally characterized for IgH and IgK expression.

Table S2. List of all gRNAs used in this study.

gRNA ID	Target region	gRNA (oligonucleotide)	Resident plasmid
gRNA-A	WEN1.3 leader-VH intron	GCTGTCGGGAGAAAGAAATTG	pX458
gRNA-B	WEN1.3 leader-VH intron	GCCCTATCTCCTCTTCAGAT	pX458
gRNA-C	WEN1.3 leader-VH intron	GTTCCAATCTGAAGAGGAGAT	pX458
gRNA-D	WEN1.3 JH downstream intron	GGAGCATGACGGACTAATCT	pX458
gRNA-E	WEN1.3 JH downstream intron	GTTGGTTTTAGCGGAGTCCC	pX458
gRNA-F	WEN1.3 VK leader	GGAGAAGCAGGACCCATAGC	pX458
gRNA-G	WEN1.3 VK leader	GGCTATGGGTCCTGCTTCTC	pX458
gRNA-H	WEN1.3 JK downstream intron	GGGATCTTCTATTGATGCAC	pX458
gRNA-I	WEN1.3 JK downstream intron	GTGGCTAAATGAGCCATTCC	pX458
gRNA-J	mRuby2	GTCATGGAAGGTTCGGTCAA	pX458.2 (BFP)
gRNA-K	mRuby2	GCATGCCGTTGATCACCGCC	pX458.2 (BFP)

Table S3. Flow cytometry labeling reagents with their working concentrations.

Target antigen	Working concentration	Dilution from stock	Incubation volume	Fluorophore	Product ID
**IgG2C**	3.3 µg/ml	1:150	100 µl	Allophycocyanin (APC)	115-135-208 (Jackson ImmunoResearch)
**IgK**	2.5 µg/ml	1:80	100 µl	Brilliant Violet 421™	409511 (BioLegend)
**Hen egg lysozyme**	0.99 µg/ml	1:62.5	100 µl	AlexaFluor®* 647	62971-10G-F (Sigma-Aldrich)

Lysozyme from hen egg white was labeled in-house with the AlexaFluor®* 647 Protein labeling kit (Molecular Probes™, Thermo Fisher Scientific, A-20173). A stock solution concentrated 61.8 µg/ml was used for dilution.

Table S4. Genotyping primers.

Primer name	Amplified region	Sequence
IgH-promoter-for	IgH locus (gDNA)	5'-GATGCTTTTCCTCAGGGAGGATTATG-3'
IgH-ext-rev	IgH locus (gDNA)	5'-TGATTGACAAGAATTTTGGACATTTAAAAAAATGAG-3'
IgK-upstream-for	IgK locus (gDNA)	5'-AGAGACAGGAGGATCTGGTCTATAAAATGA-3'
IgKJ5-rev	IgK locus (gDNA)	5'-CACCGAACGTGAGCCACAGTG-3'
HEL23-VK-leader-for	Spliced HEL23 antibody construct (cDNA)	5'-GGTGCAGATTTTCAGCTTC-3'
HyHEL10-VK-leader-for	Spliced HyHEL10 antibody construct (cDNA)	5'-TTTCACACCTCAGATACTTGG-3'
2G4-VK-leader-for	Spliced 2G4 antibody construct (cDNA)	5'-GTGTGCCCACTCAGGTC-3'
4G7-VK-leader-for	Spliced 4G7 antibody construct (cDNA)	5'-CCTGGGGTTGCTGCTG-3'
IgG2C-rev	Spliced antibody constructs (cDNA)	5'-GTTGTACCTCCACACACAG-3'

Primers utilized for PCR on genomic DNA and RNA/cDNA.
